# Developing a mHealth Routine Outcome Monitoring and Feedback App (“SMART Track”) to Support Self-Management of Addictive Behaviours

**DOI:** 10.3389/fpsyt.2021.677637

**Published:** 2021-06-18

**Authors:** Alison K. Beck, Peter J. Kelly, Frank P. Deane, Amanda L. Baker, Leanne Hides, Victoria Manning, Anthony Shakeshaft, Joanne Neale, John F. Kelly, Rebecca M. Gray, Angela Argent, Ryan McGlaughlin, Ryan Chao, Marcos Martini

**Affiliations:** ^1^Faculty of the Arts, Social Sciences and Humanities, School of Psychology, University of Wollongong, Wollongong, NSW, Australia; ^2^Illawarra Health and Medical Research Institute, University of Wollongong, Wollongong, NSW, Australia; ^3^School of Medicine and Public Health, University of Newcastle, Callaghan, NSW, Australia; ^4^Centre for Youth Substance Abuse Research, Lives Lived Well Group, School of Psychology, University of Queensland, St Lucia, QLD, Australia; ^5^Eastern Health Clinical School, Faculty of Medicine, Nursing and Health Sciences, Monash University, Melbourne, VIC, Australia; ^6^National Drug and Alcohol Research Centre, University of New South Wales, Randwick, NSW, Australia; ^7^Addictions Department, Institute of Psychiatry, Psychology & Neuroscience, King's College London, London, United Kingdom; ^8^Harvard Medical School, Harvard University, Boston, MA, United States; ^9^Centre for Social Research in Health, Faculty of Arts and Social Sciences, UNSW Sydney, Sydney, NSW, Australia; ^10^SMART Recovery Australia, Pyrmont, NSW, Australia; ^11^GHO, Customer Experience Agency, Sydney, NSW, Australia

**Keywords:** addiction, SMART recovery, routine outcome monitoring, mHealth, person-based approach, behavioral intervention technology

## Abstract

**Background:** Routine outcome monitoring (ROM) has been implemented across a range of addiction treatment services, settings and organisations. Mutual support groups are a notable exception. Innovative solutions are needed. SMART Track is a purpose built smartphone app designed to capture ROM data and provide tailored feedback to adults attending Australian SMART Recovery groups for addictive behaviour(s).

**Objective:** Details regarding the formative stage of app development is essential, but often neglected. Improved consideration of the end-user is vital for curtailing app attrition and enhancing engagement. This paper provides a pragmatic example of how principles embedded in published frameworks can be operationalised to address these priorities during the design and development of the SMART Track app.

**Methods:** Three published frameworks for creating digital health technologies (“Person-Based Approach,” “BIT” Model and IDEAS framework) were integrated and applied across two stages of research to inform the development, design and content of SMART Track. These frameworks were chosen to ensure that SMART Track was informed by the needs and preferences of the end-user (“Person-Based”); best practise recommendations for mHealth development (“BIT” Model) and a collaborative, iterative development process between the multi-disciplinary research team, app developers and end-users (IDEAS framework).

**Results:** Stage one of the research process generated in-depth knowledge to inform app development, including a comprehensive set of aims (clinical, research/organisation, and usage); clear articulation of the target behaviour (self-monitoring of recovery related behaviours and experiences); relevant theory (self-determination and social control); appropriate behavioural strategies (e.g., behaviour change taxonomy and process motivators) and key factors that may influence engagement (e.g., transparency, relevance and trust). These findings were synthesised into guiding principles that were applied during stage two in an iterative approach to app design, content and development.

**Conclusions:** This paper contributes new knowledge on important person-centred and theoretical considerations that underpin a novel ROM and feedback app for people with addictive behaviour(s). Although person-centred design and best-practise recommendations were employed, further research is needed to determine whether this leads to improved usage outcomes.

**Clinical Trial Registration:** Pilot Trial: http://anzctr.org.au/Trial/Registration/TrialReview.aspx?id=377336.

## Introduction

Routine Outcome Monitoring (ROM), or the systematic, repeated assessment of client progress throughout treatment (1), is an integral component of evidence-based healthcare for a range of chronic conditions (2), including addictive behaviours (3, 4). Traditionally, ROM has been performed using clinician-rated instruments (1). Over recent years, the importance of capturing the client perspective has been acknowledged (5). ROM data is important for treatment planning and quality assurance (6–9) and allows organisations to understand, evaluate and improve service delivery (9, 10). From a research perspective, ROM data provides insight into which clients receive the most benefit from services and how variations in care may impact engagement and outcomes (9).

Meta-analytic and/or systematic reviews largely report ROM has positive clinical benefits (1, 11, 12). Feedback from ROM assessments (rather than the assessment process in and of itself) may be central to these benefits (13), although further evidence is needed (14). Positive outcomes are more likely when feedback is immediate, personalised, delivered in an engaging and collaborative way and reflects change over time (13, 15). The vast majority of existing ROM approaches provide clinician feedback only (16, 17). This is unfortunate as direct client feedback may enhance the positive impact of ROM on treatment outcomes (1). Improving client involvement in the feedback process is an important clinical and research priority (18).

Various approaches to ROM have been implemented across a range of mental health (19) and addiction (20, 21) treatment services, settings and organisations. Mutual support groups are a notable exception. “Mutual support” refers to the reciprocal provision of social, emotional and informational support by group members undergoing recovery from addiction (22). Although accumulating evidence points to the importance and benefit of participating in mutual support (23–25) a major limitation in developing a strong evidence base has been the lack of systematic outcome data evaluating routine service provision. Although 12-step models are traditionally the most well-known and accessed model of mutual support (26), other approaches (e.g., SMART Recovery) are gaining momentum. SMART was originally developed as an alternative to 12-step approaches, with the major distinction being that it employs principles and strategies from cognitive behavioural therapy (CBT) and motivational interviewing [MI (27)]. Furthermore, unlike other clinically endorsed (3, 6) models of mutual support for addictive behaviours (e.g., 12-step approaches), SMART Recovery groups utilise a trained facilitator to oversee the conduct of the group. This provides a unique opportunity for encouraging ROM and feedback as a standard component of the groups.

Integration of ROM and feedback into clinical services is not without challenges (28, 29). Factors that can undermine engagement with ROM and feedback include the “time burden” associated with completing, scoring, interpreting or discussing outcome assessments (28, 30). Scepticism regarding the perceived relevance of the outcome(s) assessed and/or feedback generated has also been noted (31, 32). Within addiction services, client engagement, retention and follow-up are well-documented challenges (33), therefore the introduction of ROM instruments must be brief and the turn-around of feedback rapid (13). Few of the validated tools developed to assess treatment outcome within drug and alcohol settings [e.g., Addiction Severity Index (34)], have been investigated within the context of ROM and feedback (13). The utility of these instruments for continuous monitoring and feedback is therefore unknown. Furthermore, the principles of person centred care and recovery oriented service provision (35) would suggest that ROM should be holistic and multi-dimensional (36). Given the importance of brevity, this further complicates the process of instrument selection.

The idea of using technology to track progress within healthcare settings is not new but current approaches are limited (37), and further research (38) and innovation (39, 40) is needed. Unlike other Health Information Technology approaches (e.g., web-based platforms), mobile health applications (mHealth apps) have the potential to offer a quick, easy, interactive and engaging platform for tracking and accessing information about health and health-related behaviours (41). As smartphone ownership and usage is commonplace (42) smartphone apps have the added benefit of engaging individuals in real-time, in everyday situations and offering moment-to-moment, tailored content and support as needed (43). Immediacy of feedback and personalisation have been identified as factors that may increase responsiveness and ongoing motivation to utilise ROM systems (44). Accordingly, the functional capabilities of a smartphone app makes it the ideal platform to administer ROM and feedback, not only for putting the client at the centre of the ROM and feedback process, but as an engaging and streamlined mechanism for providing timely and personalised feedback across time.

Recent systematic reviews of digital recovery support services (45), digital measurement feedback systems (37, 38) and addiction-related mHealth apps [e.g., (46–49)] point to the promise of utilising technology for the purposes of ROM and feedback. However, a key limitation is the ever-increasing gap between availability of mHealth apps and scientific validation (47, 50–52). It is not surprising then that an international workshop of experts in digital health identified improved evaluation of digital health technologies as a key priority for future development efforts (53). Central to this is an improved focus on theory-informed content and design (53).

Improved attention to user engagement is also warranted, as mHealth apps are characterised by high levels of attrition. According to recent figures, 66% of app users drop out after the first use and 80% drop out within the first month (54). Inadequate consideration of the needs and preferences of the end user throughout the development process has been implicated (53, 55, 56). Fortunately, there are now several frameworks (55, 57, 58) available to ensure that app development is grounded in a sound understanding of the end user; incorporates theoretically-informed and evidence based content; and employs best practise methods for app development.

### Objectives and Importance

Details regarding the formative stage of mHealth development are essential (59, 60), but often neglected in published reports (53, 61). Improved consideration of the end user throughout development is vital for curtailing attrition and enhancing engagement (55, 56). Ensuring that appropriate theory is used to guide development is recommended for both mHealth apps (51, 53) and ROM and feedback systems (18, 37). This paper describes how published guidelines were used in the current study to ensure that these priorities were addressed throughout the development of a ROM and feedback mHealth app (“SMART Track”) for people attending Australian SMART Recovery groups (online and/or face to face). The preliminary evaluation of this app using a pilot study with nested qualitative evaluation and economic analysis is reported separately (62, 63).

## Materials and Methods

This paper addresses development items from the CONSORT E-Health (60) and mERA (59) checklists. This study has been approved by the University of Wollongong and Illawarra Shoalhaven Local Health District (ISLHD) Health and Medical Human Research Ethics Committee (2018/099; HREC/18/WGONG/34).

### Summary of Frameworks Utilised

The development of the SMART Track app was informed by three published frameworks that offer guidance on the creation of digital health technologies. Although each model can be used in isolation, we chose to combine these approaches to ensure that app development was informed by a more comprehensive set of guidelines that included foci related to the end-user [i.e., “person”; “Person-Based” (55)], best practise recommendations for mHealth development [“BIT” Model (57)] and a collaborative, iterative development process between the research team, app developers and participants [IDEAS framework (58)].

Firstly, from a person based perspective, app design, development, and content was informed by an in-depth understanding of SMART Recovery participants and the associated context/environment in which SMART Track will be used (55). Central to this approach was the conduct of preparatory qualitative work and the development of “guiding principles” which ensured that key decisions (e.g., including or rejecting app features and/or content) remained faithful to the understanding of the person, their context and the design objectives (55). Secondly, the BIT-Model represents a mechanism for improving the success with which researchers and developers are able to translate the clinical aim(s) of an intervention into an “implementable” technological solution (57). It was therefore utilised in the current study to ensure that central theoretical and technological considerations were adequately addressed. Finally, the IDEAS framework is designed to enhance the quality, relevance and likely efficacy of digital health interventions (58). It arose from a multi-disciplinary collaboration between researchers, designers and engineers and capitalises upon the strengths from each discipline by integrating behavioural theory, human-centred design and rigorous evaluation (58). Guidance pertaining to the formative stages of app development (“Integrate” and “DEsign”) were employed. It is beyond the scope of the current paper to discuss these frameworks in detail. Rather, we focus on how they were used to inform the design, content and development of SMART Track.

### Integrating the Person-Centred, BIT-Model and IDEAS Framework

**Figure 1:**
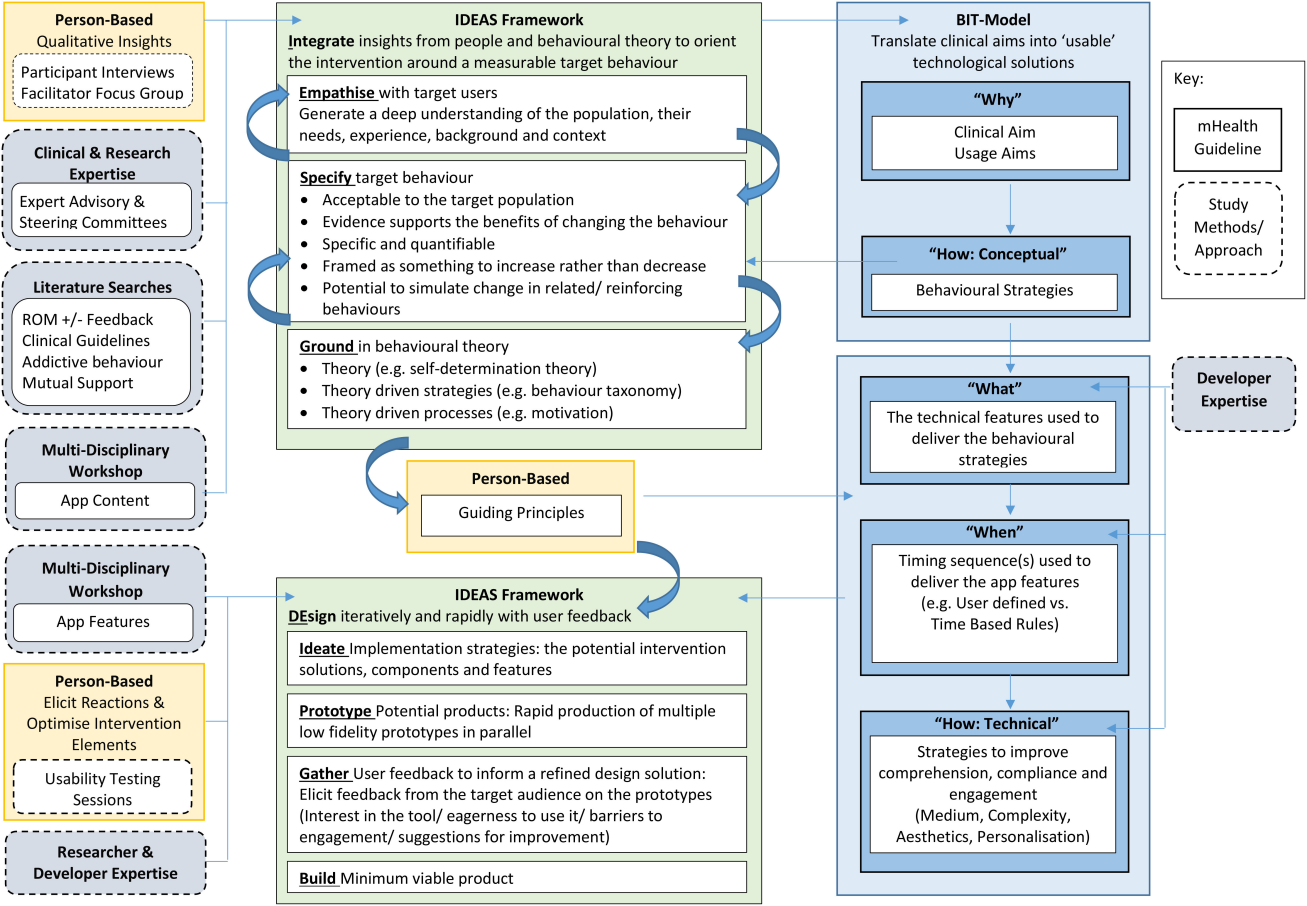
Integration of Person-Based, BIT Model, and Ideas Framework.

Our approach to integrating the three frameworks in the current study is described detail in Figure 1, and summarised below.

#### Stage One: In-depth Understanding

The initial stage of development focused on generating an in-depth understanding of people (i.e., SMART Recovery participants and SMART Recovery group facilitators), theory and behavioural strategies to guide app development. Stage one of development was informed by the guidance offered by the “Why” and “How (Conceptual)” stages of the “BIT Model” (64) and the “Integrate” phase of the IDEAS framework (58). These insights were synthesised into a set of guiding principles [person-based (55)] to inform app development, content, and design.

##### Procedure

The initial stage of development was an iterative process informed by: (a) qualitative feedback from SMART Recovery participants and SMART Recovery group facilitators; (b) clinical and research expertise from the expert advisory and steering committees; (c) a multi-disciplinary workshop facilitated by the customer experience agency contracted for app development and design (GHO, Sydney); and (d) key findings from the literature.

##### Qualitative Insights

Qualitative insights were derived via a series of 1:1 semi-structured telephone interviews with a sample of SMART Recovery participants (*N* = 20) and a semi-structured telephone focus group conducted with a sample of SMART Recovery group facilitators (*N* = 8). To allow rapid turn-around of feedback, at the end of each interview the qualitative researcher generated a brief summary of interview content (focusing on recommendations for app content, and barriers and facilitators to app use). A detailed account of the qualitative evaluation has been reported separately (65).

##### Clinical and Research Expertise

The expert advisory committee (*n* = 12) comprised researchers, clinicians, staff affiliated with SMART Recovery and/or Universities across the states of New South Wales, Queensland, and Victoria in Australia. The expert advisory committee initially met via tele/video conference fortnightly, and then monthly as the study progressed. The steering committee (*n* = 8) comprised members of the research team (chief investigator, principal investigator at SMART Recovery and trial-coordinator) and representatives from key health, non-government and Aboriginal Community Controlled organisations. Steering committee meetings were scheduled at least twice per year via tele/videoconference. Members of the expert advisory and steering committees also provided additional feedback via email and/or phone between meetings as needed.

##### Multi-Disciplinary Workshop

The multi-disciplinary workshop was facilitated by customer experience agency GHO at their Sydney office and attended by members of the development team (Executive Strategy Director, Senior UX/UI Designer, Front End Developer, Executive Creative Director, Project Manager) and expert advisory committee (researchers, clinicians and/or SMART Recovery group facilitators). Group discussion explored the objectives of the project (for the ROM and feedback tool, research team, funding body and SMART Recovery); proposed intervention features; factors that could influence engagement; and examples of how technology can be used to increase appeal/engagement with self-monitoring.

##### Key Findings From the Literature

The trial-coordinator in collaboration with members of the expert advisory committee regularly reviewed the published literature throughout the project to keep abreast of key findings pertaining to ROM and feedback (development, evaluation, engagement, tools, strategies, and/or content). Literature searches were pragmatic and ongoing as a systematic review of the literature was beyond the resources of the current project.

#### Stage Two: App Development

Stage two focused on applying the learnings from stage one (described below under results) to the design, content and development of the SMART Track app.

##### Procedure

Development [DEsign phase (58)] proceeded according to an iterative process informed by the outcomes from stage one; the digital user experience (UX) and creative expertise of GHO; and feedback from the research team, SMART Recovery participants and SMART Recovery group facilitators. Accordingly, the features (“what”), timing (“when”), delivery, design, and content (“how”: technical) of SMART Track evolved over time in-line with feedback received.

##### Multi-Disciplinary Workshop

A second multi-disciplinary workshop was held to refine the proposed content and features of the ROM and feedback (based on the learnings from stage one). This workshop was again facilitated by GHO and attended by members of the development team (Senior UX/UI Designer, Front End Developer, Executive Creative Director, Project Manager) and expert advisory committee (researchers, clinicians, and/or SMART Recovery facilitators).

##### Prototyping and Feedback

App development was broken into three “sprints,” each focusing on the design and functionality of a discrete section of the app. For each sprint, GHO used the software platforms “Sketch” to create high fidelity designs and “InVision” to present prototypes for feedback. Prototypes were presented to the research team in the first instance (via the video conferencing software “Zoom”) and amended following collaborative input from GHO and the research team.

Amended prototypes were then reviewed during nine individual usability testing sessions (three sessions per “sprint”). Participants comprised a convenience sample of SMART Recovery participants (*n* = 5), SMART Recovery group facilitators (*n* = 3), and the research team (*n* = 1). Sessions were facilitated by the Senior UX/UI Designer at GHO in collaboration with the GHO Project Manager and/or Research trial-coordinator. Within each session the participant was asked to use the prototype (via Smart phone or Web), and notes were taken about how the participant interacted with the prototype (e.g., facial expressions/body language conveying confusion and/or excitement) and any comments made. Participants were given time for spontaneous use and were also prompted with various scenarios (e.g., imagine that you wanted to add a personal motivation for change—where would you go?). The prototype was then amended in line with the verbal and non-verbal feedback collected and re-presented to the research team for final discussion, feedback, amendments and sign-off.

#### Information Synthesis

Following the methods outlined above, stage one findings were derived from the qualitative interviews with SMART Recovery participants and the focus group with SMART Recovery group facilitators; the clinical, research and development expertise of the research team and our collaborators; and a thorough understanding of relevant literature. Information was synthesised using the following methods. Content summaries generated by the qualitative researcher were reviewed by the trial-coordinator. Information pertaining to usability and acceptability was extracted. This information was summarised alongside knowledge derived from the literature and used to facilitate team discussion pertaining to app content and features. This in turn was used to generate content for each of the key considerations detailed within the Person-Centred, BIT-Model and IDEAS Framework, which was further reviewed and discussed until consensus was reached. Stage two findings were derived from applying stage one findings to the design, development and content of the SMART Track app.

## Results

### Stage One: In-depth Understanding

**Figure 2:**
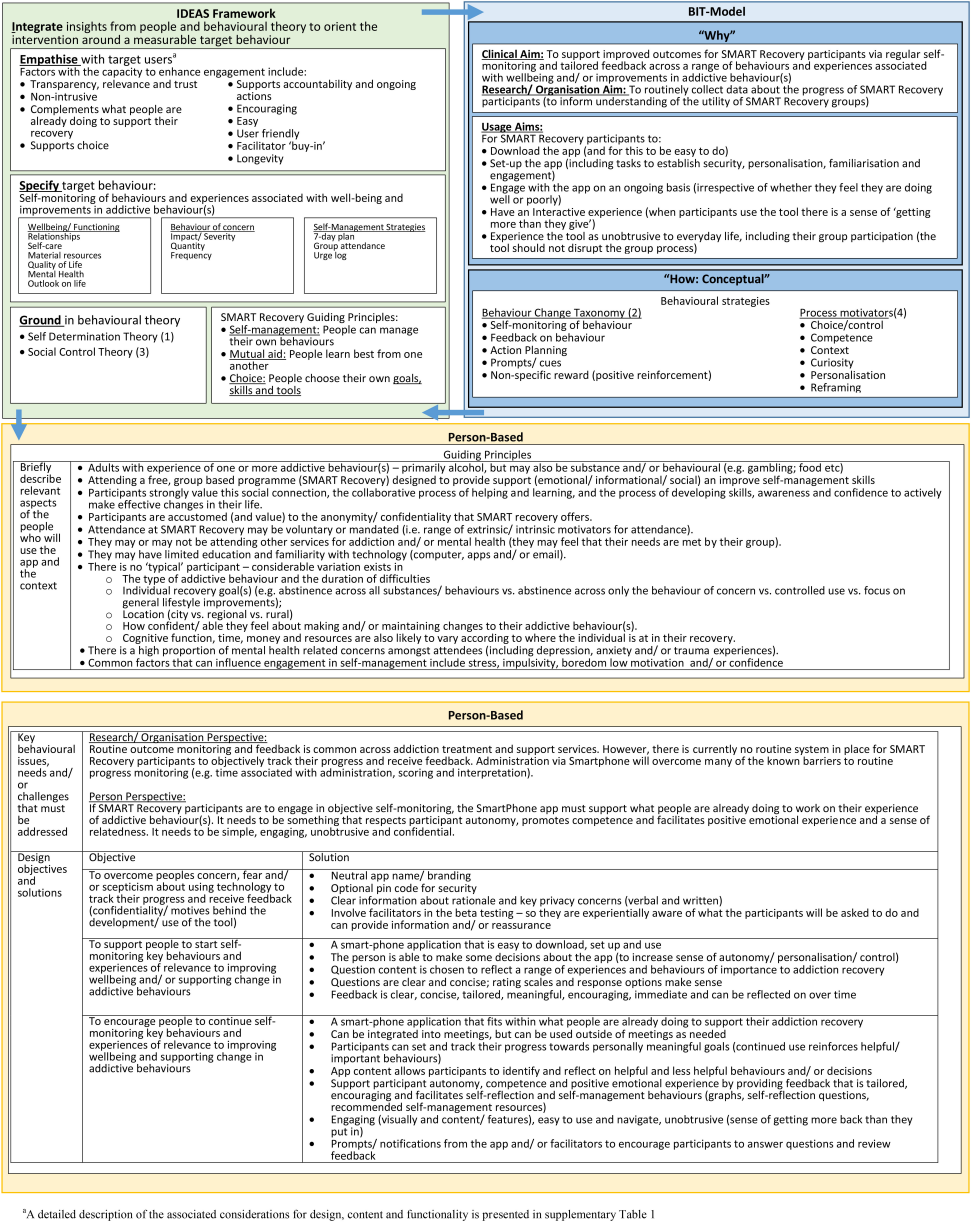
Stage one findings used to inform app development, as a function of theoretical model.

The “in-depth understanding” used to inform the development of SMART Track is described in detail in Figure 2 and summarised below according to the relevant components of each framework. Stage one culminated with the development of a set of “guiding principles” (“person based” (55) See Figure 2].

#### BIT Model: “Why”

From a funding perspective, the project remit was to develop a ROM and feedback (i.e., self-monitoring) app. From a clinical perspective, the aim was to develop a tool that participants would experience as useful in supporting their recovery (clinical aim). If it was experienced as useful, it seemed likely that participants would be more willing to use the tool beyond the initial download (usage aims), which was important as regular participant engagement with ROM and feedback represents an important opportunity for improving understanding of participant outcomes within SMART Recovery (research/organisation aim).

#### IDEAS Framework: “Empathise”

Key factors with the capacity to influence participant engagement (thereby perceived utility) of the app are outlined in Figure 2 (see also Supplementary Table 1). Participant feedback and research findings highlighted that trust in the app is of paramount importance. From this it was concluded that the rationale for data collection (and how data will be used, stored, and protected) must be made clear and the relevance to the individual highlighted. The development work also highlighted the importance of ensuring that the app is experienced as unobtrusive and that it both complements and reinforces what people are already doing to support their recovery (e.g., group attendance, working towards meaningful goals, self-management skills). Furthermore, it must be “easy” (to download, set up, navigate and use), user-friendly (e.g., non-stigmatising language, language that is simple, concrete, confident and helpful) and encouraging (e.g., “celebrate successes”). As SMART Recovery group facilitators are central to the conduct of groups, it became apparent that participant engagement may be compromised if the app is perceived by facilitators as unhelpful or disruptive. To support the longevity of the app, team discussions and expert input highlighted the importance of developing the app with future platforms in mind.

#### IDEAS Framework: “Specify”

The behaviour of interest targeted by the ROM and feedback app is regular self-assessment of progress. To enhance the perceived relevance and utility of the app to SMART Recovery participants our preparatory work demonstrated that the target content for self-assessment must be experienced by participants to be meaningful. Accordingly a range of behaviours and experiences were included to reflect the diversity of SMART Recovery participants. ROM questions were drawn from validated self-report instruments [e.g., Substance Use Recovery Evaluator (66); K-10 (67, 68); Screener for Substance and Behavioural Addictions (69)]; and as needed, wording was modified in line with participant feedback. Further details are available in the published protocol (63).

#### IDEAS Framework: “Ground”

To optimise the likelihood that participants would be willing to engage in regular self-assessment, this project drew from the program principles of SMART Recovery Australia (SRAU) (70) and insights from Self Determination Theory (71) and Social Control Theory (72). Specifically, we sought to ensure that the app complemented what participants were already doing to support their recovery. Accordingly, guided by the program principles of SRAU, SMART Track was developed so that it could be used alongside SMART Recovery group participation as a way of enhancing competence and confidence (“self-management”); facilitating meaningful discussion with peers (“mutual aid”) and supporting individual recovery goals (“choice”). Consistent with these principles, Self-Determination Theory highlights the importance of utilising competence, positive emotional experience and autonomy to motivate desired behaviour(s) (71). Finally, drawing from Social Control Theory, as connexion with others motivates “desirable” behaviour [e.g., via modelling and accountability (72)] we also sought to include features that would support connexion with and learning from others (e.g., lived experience storeys and prompts for participants to discuss their feedback at their SMART Recovery group).

#### BIT Model: “How (Conceptual)”

The preparatory work also identified “self-monitoring” and “feedback” (64) as key strategies for facilitating participant engagement in self-assessment. Additional strategies drawn from the Behaviour Change Taxonomy [e.g., action planning, prompts/cues and positive reinforcement (73)] and process motivators [e.g., strategies to facilitate choice, competence and personalization (58)] were also identified as potential ways of enhancing engagement with the process of self-monitoring.

### Stage Two: App Development

#### BIT Model: “What”

**Figure 3:**
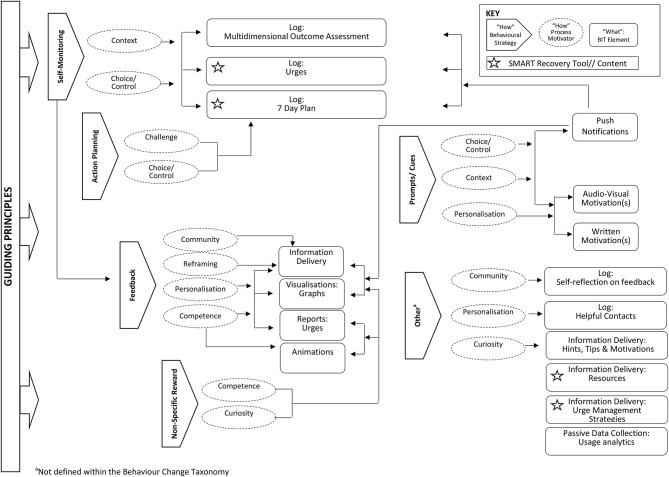
Relationship between app features and associated behavioural strategies.

The features included in SMART Track [“What”: BIT Elements (57)] are presented in Figure 3 according to the underlying behavioural strategies and process motivators [“How”: Conceptual (57)]. The relationship between these features and learnings from stage one are summarised below.

##### Features to Support Individual Recovery Journey

To enhance compatibility between SMART Track and what participants already do to support their recovery, several SMART Recovery tools are featured. Firstly, a “7-day plan” allows participants to specify details of an action plan and record their progress towards personally meaningful goals. Secondly, an interactive “urge log” allows participants to rate the strength of an urge as it is experienced, receive coping strategies and motivational messages to assist with urge management and to record associated triggers, outcome(s) and any (un)helpful management strategies. Additional SMART Recovery programme content and self-management tools are also available through a “resources” section. Participant expertise in self-management is acknowledged by including “hints, tips and motivations” derived directly from qualitative interviews with SMART Recovery participants and “motivational stories” of self-management and/or recovery.

##### Features to Respect Participant Autonomy

Respect for participant autonomy is made via several features that promote choice and control. For example, to allow people to monitor personally meaningful outcomes the app includes both multidimensional outcome assessment, and the option of tracking progress towards personally meaningful goals. There is also some flexibility in the timing and frequency of push notifications. Furthermore, the wording of push notifications (e.g., “log now,” “log later”) is such that ultimately it is up to the individual whether or when they choose to engage in the various self-monitoring tasks.

##### Features to Facilitate Positive Emotional Experience and Relatedness

When participants achieve personally meaningful goals, animations (celebratory confetti) are used to celebrate success. Progress on the various outcome assessments is highlighted and reinforced (graphs across time) and the wording of written feedback is such that all efforts are encouraged. Although a social networking feature was beyond the scope of the current app (due to funding and other pragmatic constraints), participants are encouraged to consider discussing their feedback with trusted others (e.g., SMART Recovery group). They can also use the app to log “helpful contacts” and are prompted to consider accessing support when progress is challenging and/or urges are experienced.

#### BIT Model: “When”

To balance client autonomy with routine collection of outcome data, the app includes a mix of user-defined workflows and time-based rules (64). For example, participants are prompted to link the completion of the ROM items and their 7-day plan to attendance at a SMART Recovery group; tick off their completed tasks as they are achieved and log their urges following each self-reported experience (i.e., user-defined). ROM items not completed at the end of each 1-week cycle (proposed ROM frequency) will count as “missing data” and the next week of progress monitoring will commence (i.e., time-based rules).

#### “How”: Technical Characteristics

Consistent with the guiding principles that were developed, and informed by the creative and technical expertise of GHO, decisions regarding medium, complexity, aesthetics and personalisation were made to optimise the likelihood that the app would be experienced as simple, engaging, unobtrusive and confidential.

##### Medium

Text was the primary medium employed to communicate information within the app. Given the diverse experience of SMART Recovery participants, several strategies were employed to maximise accessibility. Firstly, where possible, written information was supplemented by visual prompts (e.g., when orienting people to the app) or content (e.g., when providing feedback). Efforts were made to convey content using simple, brief sentences and a conversational tone. Where detailed information was required (e.g., privacy policy), key elements were summarised in the app, with more detailed content accessible via a link.

##### Complexity

To provide context and assist with completion, ROM items are prefaced by a brief description of item content and the rationale behind collecting this information. To simplify use, ROM items are completed using radio buttons, multi-select buttons, drop down menus and sliding scales. Free text response options are used sparingly, and restricted to capturing idiosyncratic outcomes (e.g., 7-day plan) and reflections (e.g., used to capture personal reflections on feedback received).

##### Aesthetics

The colour scheme and style of SMART Track was guided by the need to ensure that it complemented SMART Recovery branding, whilst not being instantly recognisable as to respect an individual's privacy. Otherwise, aesthetics were selected by GHO with a view to optimise ease of use (e.g., layout, interaction cues, text style, and size) and participant engagement (e.g., colour scheme, graphics).

##### Personalisation

To optimise ease of use, personal relevance and participant engagement a range of features were included to support personalisation. Participant responses to the ROM items directly inform tailored visual (graphs over time) and written feedback. Participants can elect to add one or more personal motivations for change (visual, video and/or written). This information can be amended as many times as needed and is displayed prominently within the app. A “contacts” section allows participants to enter the details of key support people. The “urge log” allows participants to enter the helpful strategies and/or motivations they would like to see when they next experience an urge.

### SMART Track: An Overview of the Resultant Mhealth ROM and Feedback App

**Figure 4:**
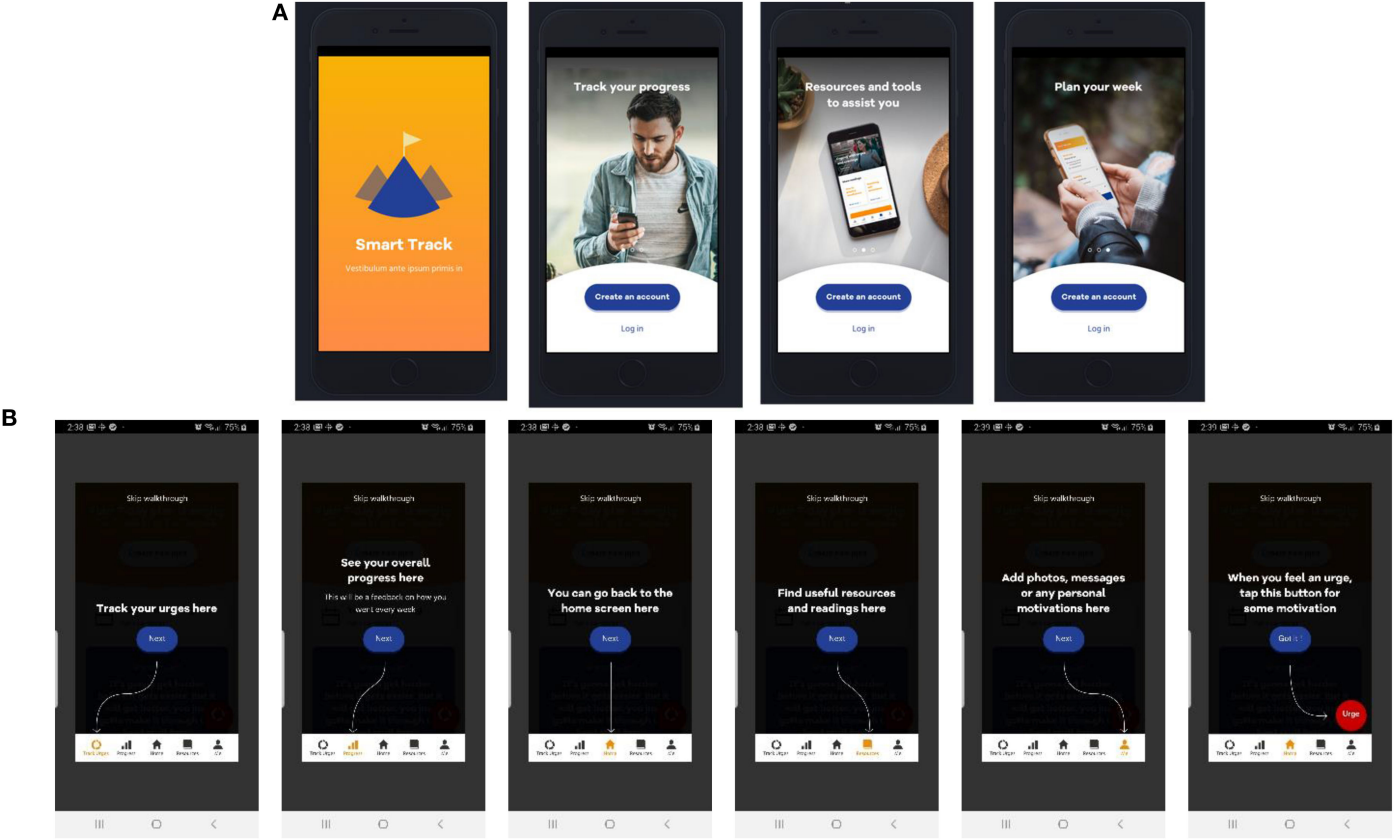
**(A)** SMART Track Overview and **(B)** Participant “Walk Through”.

**Figure 5:**
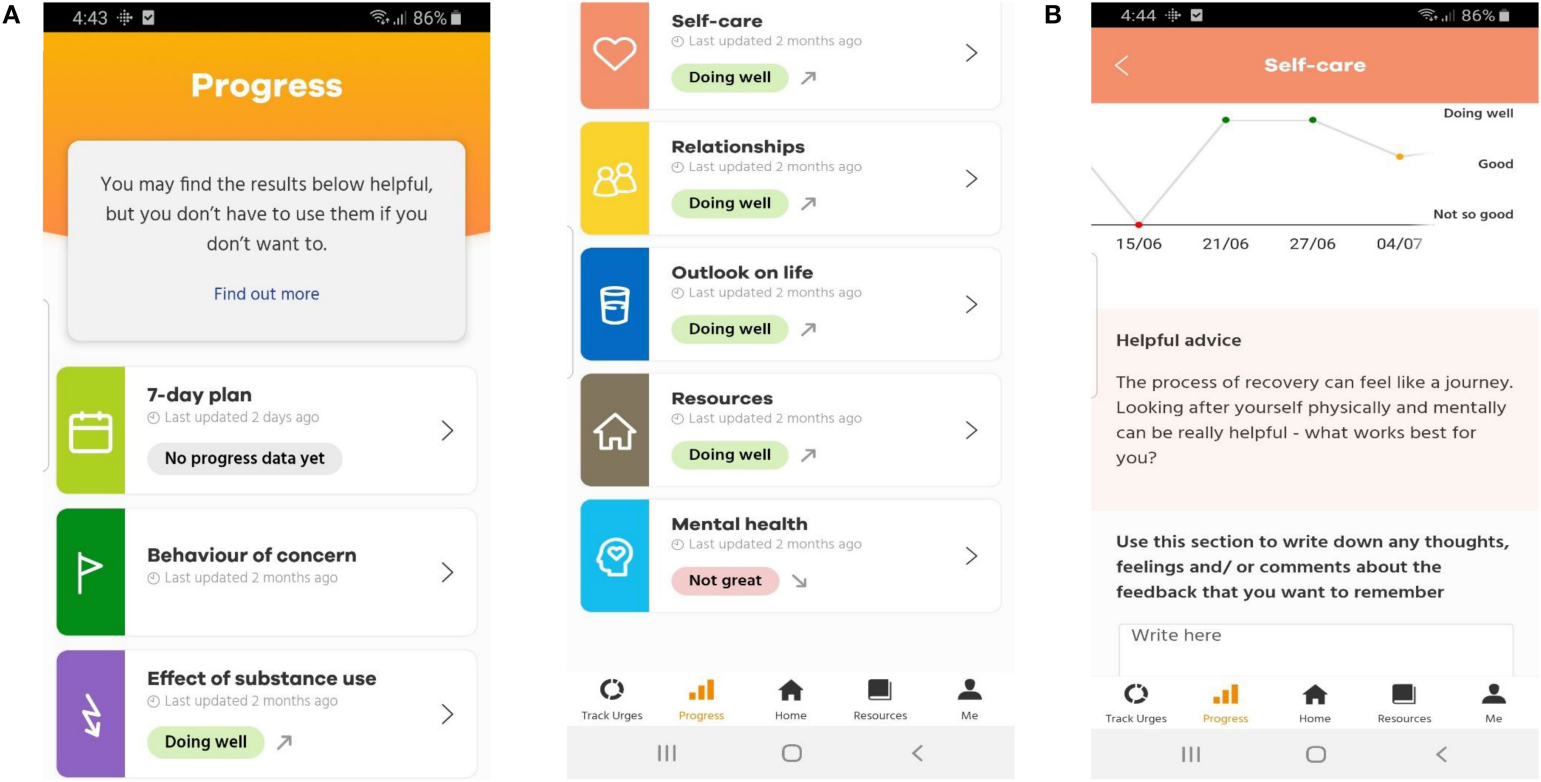
**(A)** Overall progress screen and **(B)** Progress screen for the individual domain of “self-care”.

In summary, SMART Track consists of core ROM and feedback functionality and several additional features to enhance engagement (Resources; Customisable support(s) and personal motivation(s); Interactive urge log; and Pop up motivations and self-management strategies; Figure 4). Further details are available in the published protocol (63), and briefly summarised below. SMART Track provides multi-dimensional ROM assessment and feedback (ROM items are detailed in Supplementary Table 2 as a function of target domain and assessment frequency). Feedback consists of tailored visual and written feedback across eight domains (7-day plan, behaviour of concern, effect of substance use, self-care, relationships, outlook on life, resources and mental health; Figure 5). The “Resources” screen delivers ten pieces of content. This is distributed across seven self-management resources (including SMART Recovery resources) and three motivational storeys [extracted with permission from the “Lives of Substance” website (74)]. Participants have the option of tailoring app content by uploading key contact number(s)/support services and/or personal motivation(s) for change (photo, audio, video, and/or written) into the “Me” section of the app. In addition to tracking the number, frequency and strength of urges, when the participant experiences an urge, the interactive urge log prompts participants to manage their urges, log triggers, and reflect on how to maintain/improve effective urge self-management. Participants also receive a “pop” up message when they open the app for the first time each day. SMART Track is freely available for Android (75) and Apple (75) devices.

## Discussion

This paper illustrates how three published frameworks were integrated and applied to the design and development (55, 57, 58) of an app designed to routinely monitor outcomes and provide feedback to participants attending SMART Recovery groups. It adds to the growing body of technology based solutions for ROM and feedback (37, 38, 45). By detailing the formative stage of SMART Track, this paper provides a pragmatic example of how principles embedded in these models can be operationalised to address key priorities in mHealth app development. This paper adds to the growing body of literature that has harnessed technology to overcome traditional barriers to ROM [e.g., scoring and providing tailored feedback (76)].

### Strengths

Our user-centred and theory informed approach to the design and development of SMART Track addresses key limitations (51, 53) within the mHealth literature. Evidence from mHealth apps designed to support self-management of other chronic conditions suggest that future research efforts should focus on “a simple and user-friendly-designed mHealth system, data confidentiality, lay language use for structured and automated feedback or advice, positive motivation and improving engagement” (77). When developing tools to assess patient outcomes, early, meaningful and ongoing consultation with key stakeholders is central to maximising acceptability and person engagement (78). End-users want to be actively involved and consulted throughout the development process (79). These principles were at the forefront of the current body of work.

### Challenges and Opportunities

The use of cloud functions for collecting and storing data means that SMART Track needs a reliable internet connexion to function. This is a common limitation within the digital health technology literature (37). Reliance on internet connectivity not only impacts the user experience, but may also inadvertently disadvantage certain user-groups (45). Improving offline functionality represents an important challenge for future development and evaluation efforts. Although considerable efforts were made to support a personalised experience (e.g., tailored written and visual feedback derived from participant responses; user led goal setting; choice over when/whether to receive goal-setting notifications; option to add personal motivations to support urge management) funding and time constraints meant that we were unable to action all participant priorities. For example, common requests included the option of being able to share feedback with trusted others (e.g., via shared log-in or direct export options); the option of participating in a discussion board feature (e.g., to share resources and support) and the capacity to complete self-management activities and worksheets within the app. Future research would benefit from continued efforts to improve the user experience and identify the demographic, clinical and contextual factors that may influence engagement with and utility of SMART Track. For example, by using routinely captured data analytics, and/or geospatial data SMART Track could be used to identify participant characteristics, contextual factors and/or app features associated with engagement, attrition and clinical outcomes (39, 40). Such information could have potential clinical benefits, for example providing early warning signs to participants at risk of poor outcomes, thereby allowing them to seek additional support and potentially circumvent a relapse [e.g., see (80)]. Finally, although our integration of three published frameworks ensured that the development and evaluation of SMART Track was informed by the needs and preferences of the end-user (55); best practise recommendations for mHealth development (57) and a collaborative, iterative development process (58), the process was complicated by overlap between these models. The development of new, comprehensive guidelines applicable to the development and evaluation of mHealth apps offer an important solution moving forward [e.g., The Accelerated Creation-to-Sustainment model (81)].

## Conclusions

Although the principles derived from models used in the development of SMART Track facilitated the process of app design and development, future research is needed to determine whether this leads to improved outcomes such as uptake, engagement and sustained use. One of the most critical indicators of this app's success will be the frequency that users complete the routine outcome assessments embedded in the app. Review of the extent that various features of the app are utilised will provide insights into which elements of the various models appear to facilitate user engagement. A pilot study has been conducted (62, 63) and provides preliminary evidence for the feasibility, acceptability and utility of SMART Track for ROM and feedback in SMART Recovery groups.

## Data Availability Statement

The datasets presented in this article are not readily available because the authors don't have permission to share the data. Requests to access the datasets should be directed to alisonbe@uow.edu.au.

## Ethics Statement

The studies involving human participants were reviewed and approved by the University of Wollongong and Illawarra Shoalhaven Local Health District (ISLHD) Health and Medical Human Research Ethics Committee (2018/099; HREC/18/WGONG/34). The patients/participants provided their written informed consent to participate in this study.

## Author Contributions

AKB: conceptualisation, methodology, formal analysis, investigation, data curation, writing—original draft, writing—review and editing, project administration, and funding acquisition. PK: conceptualisation, methodology, resources, writing—review and editing, supervision, and funding acquisition. FD, ALB, LH, and AS: conceptualisation, methodology, writing—review and editing, supervision, and funding acquisition. VM: conceptualisation, methodology, writing—review and editing, and supervision. JN and JK: conceptualisation, methodology, writing—review and editing, and funding acquisition. RG: methodology, formal analysis, investigation, and writing—review and editing. AA and RM: conceptualisation, methodology, resources, writing—review and editing, and funding acquisition. RC and MM: methodology, software, resources, data curation, and writing—review and editing. Authorship follows ICMJE recommendations (82). All author offered critical revisions to the manuscript for important intellectual content, have approved the final version of this manuscript, agreed to be accountable for all aspects of the work in ensuring that questions related to the accuracy or integrity of any part of the work are appropriately investigated and resolved, and made substantial contributions to study conception, design, methods, and/or the content and features of the ROM and feedback system under investigation.

## Conflict of Interest

RM is Executive Director of SMART Recovery Australia. AA is employed by SMART Recovery as the National Program Manager and Trainer. PK, FD, ALB, AS, LH, VM, AKB, JK, and AA volunteer as members of the SMART Recovery Australia Research Advisory Committee. RC is the Exec. Creative Director and MM is the BBA Technical Lead at GHO, the company contracted to design and develop the app. The potential and/or perceived conflict of interest is negligible. The role of study investigators on the research advisory committee and/or as an employee of SMART Recovery is freely available on the SMART Recovery Australia website (and study participants can be directed to this information as required). The role of GHO is the design and development of SMART Track is clearly detailed in app store description(s). Furthermore, the team responsible for informing study design and overseeing the conduct of the study and data analysis also consists of researchers, clinicians, and statisticians independent from SMART Recovery. An independent qualitative researcher was recruited to collect and analyse the qualitative data. The remaining authors declare that the research was conducted in the absence of any commercial or financial relationships that could be construed as a potential conflict of interest.

## References

[1] Carlier IV van Eden WA. Routine outcome monitoring in mental health care and particularly in addiction treatment: evidence-based clinical and research recommendations. J Addict Res Ther. (2017) 8:332. 10.4172/2155-6105.1000332

[2] Australian Health Ministers' Advisory Council. National Strategic Framework for Chronic Conditions. Australian Government (2017).

[3] National Institute for Health and Care Excellence. Alcohol-Use Disorders: Diagnosis, Assessment and Management of Harmful Drinking (High-Risk Drinking) and Alcohol Dependence. London: National Institute for Health and Clinical Excellence (2011). 39808037

[4] National Institute for Health and Care Excellence. Drug Misuse: Psychosocial Interventions. London: The British Psychological Society and The Royal College of Psychiatrists (2008).

[5] Patient Reported Measures Team. Patient Reported Measures – Program Overview. Chatswood, NSW: NSW Agency for Clinical Innovation (2018).

[6] National Institute for Health and Care Excellence. NICE Quality Standard for Drug Use Disorders. NICE Quality Standards QS23. London: National Institute in Health and Clinical Excellence (2012).

[7] National Institute for Health and Care Excellence. NICE: Alcohol-Use Disorders: Diagnosis, Assessment and Management of Harmful Drinking and Alcohol Dependence. London: National Institute for Clinical Excellence (2011). 39808037

[8] Lambert MJ Shimokawa K. Collecting client feedback. Psychotherapy. (2011) 48:72–9. 10.1037/a0022238 21401277

[9] Kelly JF Mee-Lee D. Chapter 15 - quality, accountability, and effectiveness in addiction treatment: the measurement-based practice model. In: Danovitch I, Mooney LJ, editors. The Assessment and Treatment of Addiction. St. Louis, MO: Elsevier (2019). p. 207–17.

[10] Boswell JF Constantino MJ Kraus DR Bugatti M Oswald JM. The expanding relevance of routinely collected outcome data for mental health care decision making. Adm Policy Ment Health. (2016) 43:482–91. 10.1007/s10488-015-0649-6 25861984

[11] Lambert MJ Whipple JL Kleinstauber M. Collecting and delivering progress feedback: a meta-analysis of routine outcome monitoring. Psychotherapy. (2018) 55:520–37. 10.1037/pst0000167 30335463

[12] Fortney JC Unützer J Wrenn G Pyne JM Smith GR Schoenbaum M. A tipping point for measurement-based care. Psychiatr Serv. (2016) 68:179–88. 10.1176/appi.ps.201500439 27582237

[13] Goodman JD McKay JR DePhilippis D. progress monitoring in mental health and addiction treatment: a means of improving care. Prof Psychol Res Pract. (2013) 44:231–46. 10.1037/a0032605

[14] Kendrick T El-Gohary M Stuart B Gilbody S Churchill R Aiken L. Routine use of patient reported outcome measures (PROMs) for improving treatment of common mental health disorders in adults. Cochrane Database Syst Rev. (2016) 7:CD011119. 10.1002/14651858.CD011119.pub2 27409972 PMC6472430

[15] Poston JM Hanson WE. Meta-analysis of psychological assessment as a therapeutic intervention. Psych Assess. (2010) 22:203–12. 10.1037/a0018679 20528048

[16] Davidson K Perry A Bell L. Would continuous feedback of patient's clinical outcomes to practitioners improve NHS psychological therapy services? Critical analysis and assessment of quality of existing studies. Psychol Psychother Theory Res Pract. (2015) 88:21–37. 10.1111/papt.12032 24850588

[17] Gondek D Edbrooke-Childs J Fink E Deighton J Wolpert M. Feedback from outcome measures and treatment effectiveness, treatment efficiency, and collaborative practice: a systematic review. Adm Policy Ment Health. (2016) 43:325–43. 10.1007/s10488-015-0710-5 26744316 PMC4831994

[18] Carlier IV Meuldijk D Van Vliet IM Van Fenema E Van der Wee NJ Zitman FG. Routine outcome monitoring and feedback on physical or mental health status: evidence and theory. J Eval Clin Pract. (2012) 18:104–10. 10.1111/j.1365-2753.2010.01543.x 20846319

[19] Thompson C Sansoni J Morris D Cappell J Williams K. Patient-Reported Outcome Measures: An Environmental Scan of the Australian Healthcare Sector. Sydney, NSW: Australian Commission on Safety and Quality in Health Care (2016).

[20] Kelly PJ Robinson LD Baker AL Deane FP McKetin R Hudson S. Polysubstance use in treatment seekers who inject amphetamine: drug use profiles, injecting practices and quality of life. Addict Behav. (2017) 71:25–30. 10.1016/j.addbeh.2017.02.006 28242532

[21] Kelly PJ Deane FP Baker AL Keane C. Client Outcomes Management System (COMS): Data Report, 2013. Illawarra Institute for Mental Health (2014).

[22] Public Health England. Improving Mutual Aid Engagement: A Professional Development Resource. London: Public Health England (2015).

[23] Ferri M Amato L Davoli M. Alcoholics anonymous and other 12-step programmes for alcohol dependence. Cochrane Database Syst Rev. (2006) 19:Cd005032. 10.1002/14651858.CD005032.pub2 16856072 PMC12167657

[24] Manning V Garfield JB Best D Berends L Room R Mugavin J. Substance use outcomes following treatment: findings from the Australian Patient Pathways Study. Aust N Z J Psychiatry. (2017) 51:177–89. 10.1177/0004867415625815 26769978

[25] Beck AK Forbes E Baker AL Kelly PJ Deane FP Shakeshaft A. Systematic review of SMART Recovery: outcomes, process variables, and implications for research. Psychol Addict Behav. (2017) 31:1–20. 10.1037/adb0000237 28165272

[26] Kelly JF Yeterian JD. The role of mutual-help groups in extending the framework of treatment. Alch Res Health. (2011) 33:350–5. 23580019 PMC3860535

[27] Horvath AT. Smart Recovery®: addiction recovery support from a cognitive-behavioral perspective. J Ration Emot Cogn Behav Ther. (2000) 18:181–91. 10.1023/A:1007831005098

[28] Boswell JF Kraus DR Miller SD Lambert MJ. Implementing routine outcome monitoring in clinical practice: benefits, challenges, and solutions. Psychother Res. (2015) 25:6–19. 10.1080/10503307.2013.817696 23885809

[29] Jensen-Doss A Haimes EMB Smith AM Lyon AR Lewis CC Stanick CF. Monitoring treatment progress and providing feedback is viewed favorably but rarely used in practice. Adm Policy Ment Health. (2018) 45:48–61. 10.1007/s10488-016-0763-0 27631610 PMC5495625

[30] Duncan EAS Murray J. The barriers and facilitators to routine outcome measurement by allied health professionals in practice: a systematic review. BMC Health Serv Res. (2012) 12:96. 10.1186/1472-6963-12-96 22506982 PMC3358245

[31] Solstad SM Castonguay LG Moltu C. Patients' experiences with routine outcome monitoring and clinical feedback systems: a systematic review and synthesis of qualitative empirical literature. Psychother Res. (2017) 29:157–70. 10.1080/10503307.2017.1326645 28523962

[32] Moltu C Veseth M Stefansen J Notnes JC Skjolberg A Binder PE. This is what I need a clinical feedback system to do for me: a qualitative inquiry into therapists' and patients' perspectives. Psychother Res. (2016) 28:250–63. 10.1080/10503307.2016.1189619 27219820

[33] Perkins KS Tharp BE Ramsey AT Patterson Silver Wolf D. Mapping the evidence to improve retention rates in addiction services. J Soc Work Pract Addict. (2016) 16:233–51. 10.1080/1533256X.2016.1200055

[34] McLellan AT Kushner H Metzger D Peters R Smith I Grissom G. The fifth edition of the addiction severity index. J Subst Abuse Treat. (1992) 9:199–213. 10.1016/0740-5472(92)90062-S 1334156

[35] NSW Mental Health Commission. Living Well: A Strategic Plan for Mental Health in NSW. Sydney, NSW: NSW Mental Health Commission (2014).

[36] McLellan AT McKay JR Forman R Cacciola J Kemp J. Reconsidering the evaluation of addiction treatment: from retrospective follow-up to concurrent recovery monitoring. Addict. (2005) 100:447–58. 10.1111/j.1360-0443.2005.01012.x 15784059

[37] Lyon AR Lewis CC Boyd MR Hendrix E Liu F. Capabilities and characteristics of digital measurement feedback systems: results from a comprehensive review. Adm Policy Ment Health. (2016) 43:441–66. 10.1007/s10488-016-0719-4 26860952 PMC4833592

[38] Gual-Montolio P Martínez-Borba V Bretón-López JM Osma J Suso-Ribera C. How are information and communication technologies supporting routine outcome monitoring and measurement-based care in psychotherapy? A systematic review. Int J Environ Res Public Health. (2020) 17:3170. 10.3390/ijerph17093170 32370140 PMC7246636

[39] Huckvale K Venkatesh S Christensen H. Toward clinical digital phenotyping: a timely opportunity to consider purpose, quality, and safety. Npj Digital Med. (2019) 2:88. 10.1038/s41746-019-0166-1 31508498 PMC6731256

[40] Ferreri F Bourla A Mouchabac S Karila L. e-Addictology: an overview of new technologies for assessing and intervening in addictive behaviors. Front Psychiatry. (2018) 9:51. 10.3389/fpsyt.2018.00051 29545756 PMC5837980

[41] Boudreaux ED Waring ME Hayes RB Sadasivam RS Mullen S Pagoto S. Evaluating and selecting mobile health apps: strategies for healthcare providers and healthcare organizations. Transl Behav Med. (2014) 4:363–71. 10.1007/s13142-014-0293-9 25584085 PMC4286553

[42] Drumm JW Morne NS Davey M. Mobile Consumer Survey 2017: The Australian Cut - Smart Everything, Everywhere. Sydney, NSW: Deloitte (2017).

[43] Garnett C Crane D West R Brown J Michie S. Identification of behavior change techniques and engagement strategies to design a smartphone app to reduce alcohol consumption using a formal consensus method. JMIR mHealth and uHealth. (2015) 3:e73. 10.2196/mhealth.3895 26123578 PMC4526967

[44] Taylor HE Deane F Russell B. Feasibility of using Short Message Service (SMS) to collect outcome data in an Australian residential alcohol and drug treatment service. Addict Disord Their Treat. (2018) 17:65–75. 10.1097/ADT.0000000000000125

[45] Ashford RD Bergman BG Kelly JF Curtis B. Systematic review: digital recovery support services used to support substance use disorder recovery. Hum Behav Emerg Technol. (2020) 2:18–32. 10.1002/hbe2.148

[46] Weaver ER Horyniak DR Jenkinson R Dietze P Lim MS. “Let's get Wasted!” and other apps: characteristics, acceptability, and use of alcohol-related smartphone applications. JMIR Mhealth Uhealth. (2013) 1:e9. 10.2196/mhealth.2709 25100681 PMC4114432

[47] Meredith SE Alessi SM Petry NM. Smartphone applications to reduce alcohol consumption and help patients with alcohol use disorder: a state-of-the-art review. Adv Health Care Tech. (2015) 1:47–54. 10.2147/AHCT.S65791 27478863 PMC4963021

[48] Song T Qian S Yu P. Mobile health interventions for self-control of unhealthy alcohol use: systematic review. JMIR mHealth and uHealth. (2019) 7:e10899. 10.2196/10899 30694200 PMC6371076

[49] Nesvag S McKay JR. Feasibility and effects of digital interventions to support people in recovery from substance use disorders: systematic review. J Med Internet Res. (2018) 20:e255. 10.2196/jmir.9873 30139724 PMC6127498

[50] McKay FH Cheng C Wright A Shill J Stephens H Uccellini M. Evaluating mobile phone applications for health behaviour change: a systematic review. J Telemed Telecare. (2018) 24:22–30. 10.1177/1357633X16673538 27760883

[51] Jusoh S. A survey on trend, opportunities and challenges of mhealth apps. iJIM. (2017) 11:73–85. 10.3991/ijim.v11i6.7265

[52] Singh K Drouin K Newmark LP Filkins M Silvers E Bain PA. Patient-facing mobile apps to treat high-need, high-cost populations: a scoping review. JMIR Mhealth Uhealth. (2016) 4:e136. 10.2196/mhealth.6445 27993761 PMC5206484

[53] Michie S Yardley L West R Patrick K Greaves F. Developing and evaluating digital interventions to promote behavior change in health and health care: recommendations resulting from an international workshop. JMIR. (2017) 19:e232. 10.2196/jmir.7126 28663162 PMC5509948

[54] Research2Guidance. mHealth Developer Economics: How mHealth App Publishers Are Monetizing Their Apps. Berlin: Research2Guidance (2018).

[55] Yardley L Morrison L Bradbury K Muller I. The person-based approach to intervention development: application to digital health-related behavior change interventions. JMIR. (2015) 17:e30. 10.2196/jmir.4055 25639757 PMC4327440

[56] Simblett S Greer B Matcham F Curtis H Polhemus A Ferrão J. Barriers to and facilitators of engagement with remote measurement technology for managing health: systematic review and content analysis of findings. JMIR. (2018) 20:e10480. 10.2196/10480 30001997 PMC6062692

[57] Mohr DC Schueller SM Montague E Burns MN Rashidi P. The behavioral intervention technology model: an integrated conceptual and technological framework for eHealth and mHealth interventions. JMIR. (2014) 16:e146. 10.2196/jmir.3077 24905070 PMC4071229

[58] Mummah SA Robinson TN King AC Gardner CD Sutton S. IDEAS (Integrate, Design, Assess, and Share): a framework and toolkit of strategies for the development of more effective digital interventions to change health behavior. JMIR. (2016) 18:e317. 10.2196/jmir.5927 27986647 PMC5203679

[59] Agarwal S LeFevre AE Lee J L'Engle K Mehl G Sinha C. Guidelines for reporting of health interventions using mobile phones: mobile health (mHealth) evidence reporting and assessment (mERA) checklist. BMJ. (2016) 352:i1174. 10.1136/bmj.i1174 26988021

[60] Eysenbach G. CONSORT-EHEALTH: improving and standardizing evaluation reports of web-based and mobile health interventions. J Med Internet Res. (2011) 13:e126. 10.2196/jmir.1923 22209829 PMC3278112

[61] Agarwal S Lefevre AE Labrique AB. A call to digital health practitioners: new guidelines can help improve the quality of digital health evidence. JMIR Mhealth Uhealth. (2017) 5:e136. 10.2196/mhealth.6640 28986340 PMC5650671

[62] Kelly PJ Beck AK Deane FP Larance B Baker AL Hides L. Feasibility of a mobile health app for routine outcome monitoring and feedback in SMART recovery mutual support groups: a stage one mixed-methods pilot study. JMIR (Accepted). 10.2196/25217 PMC8529481 34612829

[63] Kelly PJ Beck AK Deane FP Larance B Baker AL Hides L. Feasibility of a mobile health app for routine outcome monitoring and feedback in SMART recovery mutual support groups: a stage one mixed-methods pilot study. JMIR Res Protoc. (2020) 9:e15113. 10.2196/15113 32673272 PMC7380906

[64] Mohr DC Burns MN Schueller SM Clarke G Klinkman M. Behavioral intervention technologies: evidence review and recommendations for future research in mental health. Gen Hosp Psychiatry. (2013) 35:332–8. 10.1016/j.genhosppsych.2013.03.008 23664503 PMC3719158

[65] Gray RM Kelly PJ Beck AK Baker AL Deane FP Neale J. A qualitative exploration of SMART Recovery meetings in Australia and the role of a digital platform to support routine outcome monitoring. Addict Behav. (2020) 101:106144. 10.1016/j.addbeh.2019.106144 31648139

[66] Neale J Vitoratou S Finch E Lennon P Mitcheson L Panebianco D. Development and validation of 'SURE': a patient reported outcome measure (PROM) for recovery from drug and alcohol dependence. Drug Alch Dep. (2016) 165:159–67. 10.1016/j.drugalcdep.2016.06.006 27344196 PMC4946826

[67] Kessler RC Barker PR Colpe LJ Epstein JF Gfroerer JC Hiripi E. Screening for serious mental illness in the general population. Arch Gen Psychiatry. (2003) 60:184–9. 10.1001/archpsyc.60.2.184 12578436

[68] Kessler RC Andrews G Colpe LJ Hiripi E Mroczek DK Normand SL. Short screening scales to monitor population prevalences and trends in non-specific psychological distress. Psychol Med. (2002) 32:959–76. 10.1017/S0033291702006074 12214795

[69] Schluter MG Hodgins DC Wolfe J Wild TC. Can one simple questionnaire assess substance-related and behavioural addiction problems? Results of a proposed new screener for community epidemiology. Addiction. (2018) 113:1528–37. 10.1111/add.14166 29357188

[70] SMART Recovery Australia. About the Program. Available online at: https://smartrecoveryaustralia.com.au/wp-content/uploads/2015/07/SMART-Recovery-About-the-program.pdf

[71] Deci EL Ryan RM. The “what” and “why” of goal pursuits: human needs and the self-determination of behavior. Psychological Inquiry. (2000) 11:227–68. 10.1207/S15327965PLI1104_01

[72] Moos RH. Theory-based active ingredients of effective treatments for substance use disorders. Drug Alch Dep. (2007) 88:109–21. 10.1016/j.drugalcdep.2006.10.010 17129682 PMC1896183

[73] Michie S Richardson M Johnston M Abraham C Francis J Hardeman W. The behavior change technique taxonomy (v1) of 93 hierarchically clustered techniques: building an international consensus for the reporting of behavior change interventions. Ann Behav Med. (2013) 46:81–95. 10.1007/s12160-013-9486-6 23512568

[74] National Drug Research Institute. Lives of Substance. Curtin University (2016). Available online at: https://www.livesofsubstance.org/resources-information/

[75] SMART Recovery. SMART Track Google Play. (2021). Available online at: https://play.google.com/store/apps/details?id=au.com.smartrecoveryaustralia

[76] Lewis CC Boyd M Puspitasari A Navarro E Howard J Kassab H. Implementing measurement-based care in behavioral health: a reviewmeasurement-based care in behavioral health measurement-based care in behavioral health. JAMA Psychiatry. (2019) 76:324–35. 10.1001/jamapsychiatry.2018.3329 30566197 PMC6584602

[77] Lee JA Choi M Lee SA Jiang N. Effective behavioral intervention strategies using mobile health applications for chronic disease management: a systematic review. BMC Med Inform Decis Mak. (2018) 18:12. 10.1186/s12911-018-0591-0 29458358 PMC5819153

[78] Neale J Strang J. Blending qualitative and quantitative research methods to optimize patient reported outcome measures (PROMs). Addiction. (2015) 110:1215–6. 10.1111/add.12896 25845408

[79] Lucock M Halstead J Leach C Barkham M Tucker S Randal C. A mixed-method investigation of patient monitoring and enhanced feedback in routine practice: barriers and facilitators. Psychother Res. (2015) 25:633–46. 10.1080/10503307.2015.1051163 26436605 PMC4867876

[80] Hehlmann MI Schwartz B Lutz T Gómez Penedo JM Rubel JA Lutz W. The use of digitally assessed stress levels to model change processes in CBT - a feasibility study on seven case examples. Front Psychiatry. (2021) 12:613085. 10.3389/fpsyt.2021.613085 33767638 PMC7985334

[81] Mohr DC Lyon AR Lattie EG Reddy M Schueller SM. Accelerating digital mental health research from early design and creation to successful implementation and sustainment. J Med Internet Res. (2017) 19:e153. 10.2196/jmir.7725 28490417 PMC5443926

[82] ICMJE. Recommendations for the Conduct, Reporting, Editing, and Publication of Scholarly Work in Medical Journals. International Committee of Medical Journal Editors (2018). 25558501

