# Supplementary material for: Developing a mHealth Routine Outcome Monitoring and Feedback App (“SMART Track”) to Support Self-Management of Addictive Behaviours
Source: Front Psychiatry. 2021 Jun 18;12:677637. doi: 10.3389/fpsyt.2021.677637 (PMC8249767; doi:10.3389/fpsyt.2021.677637)
Supplement: Supplementary file 1 [file Table_1.DOCX]

Supplementary Material

# Supplementary Table 1.

*Identified needs for enhancing engagement and associated considerations for design, content and functionality*

| Factor | Considerations for design, content and functionality |
| --- | --- |
|  |  |
| Transparency, relevance and trust | - Clear from the outset about what is being done and why – clear and early rationale to support engagement) - Address   - Relevance of the tool to individual recovery journey   - scepticism/ suspicion about the motives for collecting the data   - Fear about how the information will be used   - Concerns about privacy/ confidentiality |
| Non-intrusive | - to the SMART Recovery group - to their life - “get more out of it than you put in” - Feedback is immediate, objective and tied to a personally meaningful behaviour/ outcome |
| Complement what people are already doing to support their recovery | - Support familiar tools/ strategies   - ‘Seven day plan’   - Social connectedness   - Helpful decision making around addiction related behaviours - Reflect individual needs and goals - Capacity to be integrated into SMART Recovery meetings - Support group attendance (and ongoing value of helping others and learning from others) - Foster communication and collaboration (i.e. via making use of the SMART Recovery group) - Encourage social connectedness more broadly - Empower participants - Promote awareness - Facilitate self-reflection - Information (but not lecturing) - Normalisation - Use is tailored to ‘where people are at’ |
| Support choice (e.g. over goals, skills, tools, app features) | - Self-directed goals (weekly action plan) - Choice over the SMART Recovery tools utilised - Choice over which behaviour(s) to focus on - Up to the individual whether/ how they choose to act upon the feedback (including whether/ how to involve the group and facilitator) |
| Support accountability and ongoing actions | - Capacity to re-set and try again if goal(s) don’t work out - “pre-empt issues” - “keep on track” - Normative feedback/ benchmarking - Tailor the frequency/ timing of reminders to complete |
| Encouraging | - Celebrate successes - Prompt ongoing action in the face of challenges - Promote ‘stories of success’ (inspiration(s) from others/ hints/ tips etc) - use gamification to engage, motivate and promote self-awareness and behaviour change |
| Easy | - to download - to complete - to navigate - to use |
| User friendly | - Suitable for the broad community that attends SMART Recovery (different levels of literacy/ technology experience/ cognitive capacity) - Language needs to be   - non-stigmatising, non-diagnostic, non-clinical and non-judgemental,   - simple, concrete, confident and hopeful - Small sentences, use of graphics - Good graphic design - Appealing interface - Able to provide feedback about the app |
| Facilitator ‘buy-in’ | - Useful to participants - Not disrupt the group process - Ability to track group function/ facilitator style (e.g. what works well/ less well for group members; authenticity, non-judgemental stances, empathy, and a group version of a Session Rating Scale) |
| Longevity | - Compatibility with future platforms/ infrastructure - Applicability to other services/ settings offering treatment and/or support to people with experience of addictive behaviours |
