# Supplementary material for: Developing a mHealth Routine Outcome Monitoring and Feedback App (“SMART Track”) to Support Self-Management of Addictive Behaviours
Source: Front Psychiatry. 2021 Jun 18;12:677637. doi: 10.3389/fpsyt.2021.677637 (PMC8249767; doi:10.3389/fpsyt.2021.677637)
Supplement: Supplementary file 2 [file Table_2.docx]

Supplementary Table 2

Smart Track ROM items and response categories as a function of outcome domain and assessment frequency

| **Domain** | **Frequency** | **Instrument** | **Item(s)** | **Response Option(s)** |
| --- | --- | --- | --- | --- |
| 1. Goal setting | Weekly  (Weeks A and B) | Adapted from the Change Plan(1, 2) | Your 7-day plan:  Add task(s) for this week | One or more tasks are added via free text and/ or selecting from some example tasks (e.g. go for a jog, phone a friend), and the participant is prompted to set the time and date for completing each task and whether/ when they wish to set a reminder |
| 1. Values | Weekly  (Weeks A and B)  [per 7-day plan task(s) set] | Adapted from the Valued Living Questionnaire(3) | This task is important to me for  Family, Intimate relationships, Marriage, Parenting, Friendship, Work, Education, Learning, Recreation, Spirituality, Citizenship, Community involvement, Physical self-care, Psychological self-care, Other | One or more domains are selected |
| 1. Self-efficacy | Weekly  (Weeks A and B) | Confidence Ruler  (1, 2, 4) | How confident do you feel about achieving this plan? | Sliding scale from 0-10 |
| 1. Goal attainment | Weekly  (Weeks A and B) | Adapted from the Client Rating of Homework Performance(5) | Your 7-day plan: Task X | For each task, progress is indexed by selecting ‘Done’, ‘Some’ or ‘Not yet started’ |
| 1. Group attendance | Weekly  (Weeks A and B) | --- | Not counting today, how many SMART Recovery groups have you gone to in the last seven days? | 0-7 |
| 1. Frequency of addictive behaviour: Alcohol and other drug use | Weekly  (Weeks A and B) | Adapted from the COMS Questionnaire: Drug and Alcohol Use Scale(6)^a^ | Which of the following (if any) did you use this week?  Alcohol, Cannabis, Amphetamines, Benzodiazepines, Heroin, Other opioid-based drug, Cigarettes, Other (please specify), None | One or more response options are selected |
|  |  | Adapted from the COMS Questionnaire: Drug and Alcohol Use Scale(6)^a^ | How many days did you use each of the following? | Sliding scale (0-7) for each of the substances selected at item six |
| 1. Frequency of addictive behaviour: Other addictive behaviours | Weekly  (Weeks A and B) | Adapted from the Screener for Substance and Behavioural Addictions(7) | Think about the statement “I did it too much”.  In the last seven days, how often did this apply to:  Gambling, Shopping, Sex, Pornography, Internet, Food, Video-gaming, Other | Sliding scale (0-7) for each of the behaviours listed |
| 1. Quantity of alcohol use^b^ | Weekly  (Weeks A and B) | Adapted from the COMS Questionnaire: Drug and Alcohol Use Scale(6)^a^ | On average, how many standard drinks did you have on those days when you were drinking | A number from 0-100 is selected |
| 1. Quantity of cigarette use^c^ | Weekly  (Weeks A and B) | Adapted from the COMS Questionnaire: Drug and Alcohol Use Scale(6)^a^ | How many cigarettes/cigars/pipes did you have on a typical day when you did use tobacco? _____ cigarettes/cigars/pipes. | A number from 0-100 is selected |
| 1. Alcohol and drug use: Impact | Fortnightly  (Week A) | SURE: Drinking and Drug Use Subscale(8) | Items 4-6 (e.g. I have coped with problems without misusing drugs or alcohol) | ‘All of the time’, ‘Most of the time’, ‘A fair amount of the time’, ‘A little of the time’, ‘None of the time’ |
| 1. Self-care | Fortnightly  (Week A) | SURE: Self-Care Subscale(8) | Entire subscale (items 7-11; e.g. I have been taking care of my physical health) | As above |
| 1. Social support | Fortnightly  (Week A) | SURE: Relationships subscale(8) | Entire subscale (items 12-15; e.g. I have been getting on well with people | As above |
| 1. Resources | Fortnightly  (Week A) | SURE: Material Resources subscale(8) | Entire subscale (items 16-18; e.g. I have had stable housing) | As above |
| 1. Optimism | Fortnightly  (Week A) | SURE: Outlook on Life subscale(8) | Entire subscale (items 19-21; e.g. I have felt positive) | As above |
| 1. Mental health | Fortnightly  (Week B) | Kessler – Six Item Questionnaire(9) | Entire questionnaire | ‘None of the time’, ‘A little of the time’, ‘Some of the time’, ‘Most of the time’, ‘All of the time’ |
| 1. Quality of life | Fortnightly  (Week B) | EUROHIS-QOL 8-item index(10) | Item One:  How would you rate your quality of life? | ‘Very Good’, ‘Good’, ‘Neither poor nor good’, ‘Poor’, ‘Very Poor’ |
| 1. Urges | As needed | Adapted from The Urge Log ((1, 2)) | --- | Date and time are automatically captured when the participant clicks on the ‘Urge’ button |
|  |  |  | How intense is your urge? | ‘Low’, ‘Medium’, ‘Strong’ |
|  |  |  | What was going on? (e.g. think about where you were, who was there, what was around you and what you were doing) | Open text |
|  |  |  | How were you feeling?  Tired, Stressed, Hungry, Bored, Upset, Lonely, Relaxed, Happy, Excited, Angry, Worried, Frustrated, Energized, Ashamed, Other | Select one or more of the experiences listed |
|  |  |  | Were you happy with how you handled it? | Yes/ No |
|  |  |  | If yes: What worked for you this time? | Free text |
|  |  |  | If no: What would you like to do differently next time? | Free text |

*Notes^.^* COMS: Client Outcome Management System(11); SURE: Substance Use Recovery Evaluator(8);

^a^This subscale of the Client Outcome Management System is derived from the Brief Treatment Outcome Measure(12);

^b^Only if participant endorses ‘alcohol’ use over the preceding one week (item six);

^c^Only if participant endorses ‘cigarette’ use over the preceding one week (item six);

Supplementary References

1. SMART Recovery Australia. SMART Recovery Australia Participants' Manual: Tools and strategies to help you manage addictive behaviours. Wooloomooloo: SMART Recovery Australia; 2016.

2. SMART Recovery Australia. SMART Recovery Facilitator Training Manual: Practical Information and Tools to Help You Facilitate a SMART Recovery Group. Haymarket: SMART Recovery Australia; 2015.

3. Wilson KG, Sandoz EK, Kitchens J, Roberts M. The Valued Living Questionnaire: Defining and Measuring Valued Action within a Behavioral Framework. The Psychological Record. 2010;60(2):249-72.

4. Miller WR, Rollnick S. Motivational interviewing: Helping people change. 3rd ed. New York: Guildford Press; 2013.

5. Nikolaos Kazantzis FPDaKRR. Assessment of Homework Completion. In: Nikolaos Kazantzis FPD, Kevin R. Ronan and Luciano L'Abate, editor. Using Homework Assignments in Cognitive Behaviour Therapy. New York: Routledge Taylor & Francis Group; 2005. p. 50-60.

6. NADA. NGO Drug and Alcohol and Mental Health Information Management Project: Determining the Treatment Outcomes Data Collection Set. Strawberry Hills: NADA; 2009.

7. Schluter MG, Hodgins DC, Wolfe J, Wild TC. Can one simple questionnaire assess substance-related and behavioural addiction problems? Results of a proposed new screener for community epidemiology. Addict. 2018;113(8):1528-37.

8. Neale J, Vitoratou S, Finch E, Lennon P, Mitcheson L, Panebianco D, et al. Development and validation of 'SURE': A patient reported outcome measure (PROM) for recovery from drug and alcohol dependence. Drug Alch Dep. 2016;165:159-67.

9. Kessler RC, Andrews G, Colpe LJ, Hiripi E, Mroczek DK, Normand SL, et al. Short screening scales to monitor population prevalences and trends in non-specific psychological distress. Psychol Med. 2002;32(6):959-76.

10. Rocha NSd, Power MJ, Bushnell DM, Fleck MP. The EUROHIS-QOL 8-Item Index: Comparative Psychometric Properties to Its Parent WHOQOL-BREF. Value in Health. 2012;15(3):449-57.

11. Network of Alcohol & Other Drugs Agencies. Using the Client Outcomes Management System (COMS). Sydney: NSW Ministry of Health; 2012.

12. Lawrinson P, Copeland J, Indig D. The brief treatment outcome measure: opioid maintenance pharmacotherapy (BTOM) manual. Sydney; 2003.
